# Upregulated Na^+^/H^+^-Exchange Protects Human Colon Cancer Tissue against Intracellular Acidification

**DOI:** 10.1155/2019/3702783

**Published:** 2019-01-29

**Authors:** Ninna C. S. Voss, Henrik Kold-Petersen, Mikkel B. Henningsen, Casper Homilius, Ebbe Boedtkjer

**Affiliations:** ^1^Department of Biomedicine, Aarhus University, Ole Worms Allé 3, Building 1170, DK-8000 Aarhus C, Denmark; ^2^Regional Hospital Randers, Skovlyvej 15, DK-8930 Randers, Denmark

## Abstract

Increased metabolism accelerates local acid production in cancer tissue. The mechanisms eliminating acidic waste products from human colon cancer tissue represent promising therapeutic targets for pharmacological manipulation in order to improve prognosis for the increasing number of patients with colon cancer. We sampled biopsies of human colonic adenocarcinomas and matched normal colon tissue from patients undergoing colon cancer surgery. We measured steady-state intracellular pH and rates of net acid extrusion in freshly isolated human colonic crypts based on fluorescence microscopy. Net acid extrusion was almost entirely (>95%) Na^+^-dependent. The capacity for net acid extrusion was increased and steady-state intracellular pH elevated around 0.5 in crypts from colon cancer tissue compared with normal colon tissue irrespective of whether they were investigated in the presence or absence of CO_2_/HCO_3_^–^. The accelerated net acid extrusion from the human colon cancer tissue was sensitive to the Na^+^/H^+^-exchange inhibitor cariporide. We conclude that enhanced net acid extrusion via Na^+^/H^+^-exchange elevates intracellular pH in human colon cancer tissue.

## 1. Introduction

Current treatment options for colonic adenocarcinoma include surgery, radiation, and chemotherapy depending on the stage of disease but mortality rates remain considerable, particularly for patients with disseminated cancer.

Insufficient blood supply, elevated metabolism, and a shift from oxidative phosphorylation towards fermentative glycolysis acid-loads the intracellular compartment of cancer cells [1]. Despite the increased production of acidic waste in solid cancer tissue, intracellular pH (pH_i_) of cancer cells is typically maintained equal to or above that of normal cells [2, 3]. In contrast, the extracellular compartment in cancer tissue is up to one pH-unit more acidic than in corresponding normal tissue [4]. Studies propose that compartmentalized regulation of pH in cancer tissue facilitates cancer cell invasion and metastasis [5, 6], which are the prime causes of cancer mortality.

Most existing data regarding acid-base regulation in cancer cells derive from cultured cell lines and only few have looked at freshly isolated tissue. In order to exploit the therapeutic potential of acid-base transporters, it is essential to define the mechanisms of acid-base regulation in relevant human cancer tissue. In the current study, we evaluated pH_i_ regulation in freshly isolated crypts from human colon cancer and matched normal colon tissue in an attempt to reveal the mechanisms of acid-base transport in colonic adenocarcinomas and the adaptations taking place during colon carcinogenesis.

## 2. Materials and Methods

We sampled biopsies from human colon resections immediately after excision from patients undergoing colon cancer surgery at Regional Hospital Randers, Denmark [7]. Normal colon tissue was biopsied from the same surgical specimens at a minimal distance of 10 cm from the macroscopic tumor border. The sampling procedure was approved by the Mid-Jutland Regional Committee on Health Research Ethics (enquiry no. 157/2014). According to Danish legislation, written informed consent was not required because the procedures involve excess resected tissue from a surgical procedure where all postsurgical tissue and data handling were anonymized. The biopsies were placed in ice-cold DMEM F12 (Gibco, Denmark) and kept on ice during transport (~30 minutes) to the Department of Biomedicine at Aarhus University. The study included biopsy-verified adenocarcinomas from patients, who had not received radiotherapy in the area or recent chemotherapy.

### 2.1. Preparation of Colonic Crypts

Human colon crypts were prepared as previously described [8] by placing small samples of biopsy material in Ca^2+^-free Ringer solution (in mM: 130 Na^+^, 132 Cl^–^, 5 K^+^, 1 Mg^2+^, 5 pyruvate, 10 HEPES, 5 EDTA, and 5 glucose; adjusted to pH 7.4) in a 37°C water bath on a shaking table for 20 minutes. After vigorous shaking, the samples sedimented for 5 minutes before the supernatant was removed and the pellet was washed three times with DMEM.

### 2.2. Intracellular pH Measurements

Human colon crypts were loaded with 3 *μ*M BCECF-AM (Invitrogen, Denmark) in 0.1% DMSO for 20 minutes. The crypts were placed in a custom-made chamber heated to 37°C. For experiments performed in presence of CO_2_/HCO_3_^–^, bath solutions were bubbled continuously with 5% CO_2_/balance air. Loaded crypts were excited alternatingly at approximately 490 and 440 nm and emission light collected at 510 nm using a Nikon Diaphot 200 microscope (Nikon, Japan) equipped with an SRV CCD Retiga camera (QImaging, Canada) and VisiView software (Visitron systems, Germany) or using an Olympus IX70 wide-field microscope coupled to an EasyRatioPro fluorescence imaging system (Photon Technology International, USA). BCECF fluorescence ratios were calibrated to pH based on the high-[K^+^] nigericin method [9].

Intracellular acidification was achieved with NH_4_^+^-prepulse technique [10]. Intrinsic intracellular buffering capacity was calculated from the pH_i_ change induced by washout of NH_4_Cl in absence of CO_2_/HCO_3_^–^ [11]. The contribution of CO_2_/HCO_3_^–^ to intracellular buffering was calculated as 2.3 times the intracellular concentration of HCO_3_^–^ [12]. Assuming that NH_3_ and CO_2_ are in equilibrium across the cell membrane, the acid load during NH_4_^+^-prepulses and the intracellular concentration of HCO_3_^–^ were calculated from the Henderson-Hasselbalch equation. We plotted the intrinsic buffering capacities derived from individual experiments as function of the corresponding pH_i_ values calculated as the mean of the pH_i_ before and after NH_4_Cl washout. We investigated the recovery of pH_i_ from acidosis in the absence of bath Na^+^ and then after Na^+^-containing buffer was returned to the experimental chamber. The pH_i_ recovery rate was quantified during the last 60 seconds before and for three consecutive 60-second periods after readdition of bath Na^+^. Net acid extrusion was calculated as the product of the pH_i_ recovery rate and the buffering capacity corresponding to the midpoint of the evaluated pH_i_ interval. Because pH_i_ recovery was very modest when crypts were exposed to 10 *μ*M of the Na^+^/H^+^-exchange inhibitor cariporide, net acid extrusion in these experiments was quantified for a single 60-second period corresponding to the first pH_i_ recovery phase after addition of bath Na^+^ in experiments without cariporide present.

The CO_2_/HCO_3_^–^-containing buffer consisted of (in mM): 127 Na^+^, 4 K^+^, 1.6 Ca^2+^, 1.2 Mg^2+^, 111.02 Cl^–^, 22 HCO_3_^–^, 1.2 SO_4_^2–^, 1.18 H_2_PO_4_^–^, 10 HEPES, 5.5 glucose, and 0.03 EDTA. All buffers used in functional experiments contained 5 mM probenecid in order to prevent extrusion of BCECF by the organic anion transporter. In CO_2_/HCO_3_^–^-free buffers, HCO_3_^–^ was substituted with equimolar amounts of Cl^–^; and in Na^+^-free solutions, Na^+^ was substituted with equimolar amounts of* N*-methyl-D-glucammonium. All buffers were adjusted to pH 7.4 at 37°C.

### 2.3. Statistics

Data are expressed as mean ± SEM and* n* equals number of patients. To test the effect of two variables on the measured variable, we performed two-way ANOVA followed by Sidak's posttest. We compared cellular net acid extrusion and buffering capacity as function of pH_i_ between normal and cancer tissue using least-squares linear regression analyses. A probability (*P*) value below 0.05 was considered statistically significant. Statistical analyses were performed using GraphPad Prism 7.03 software (USA).

## 3. Results

**Figure 1:**
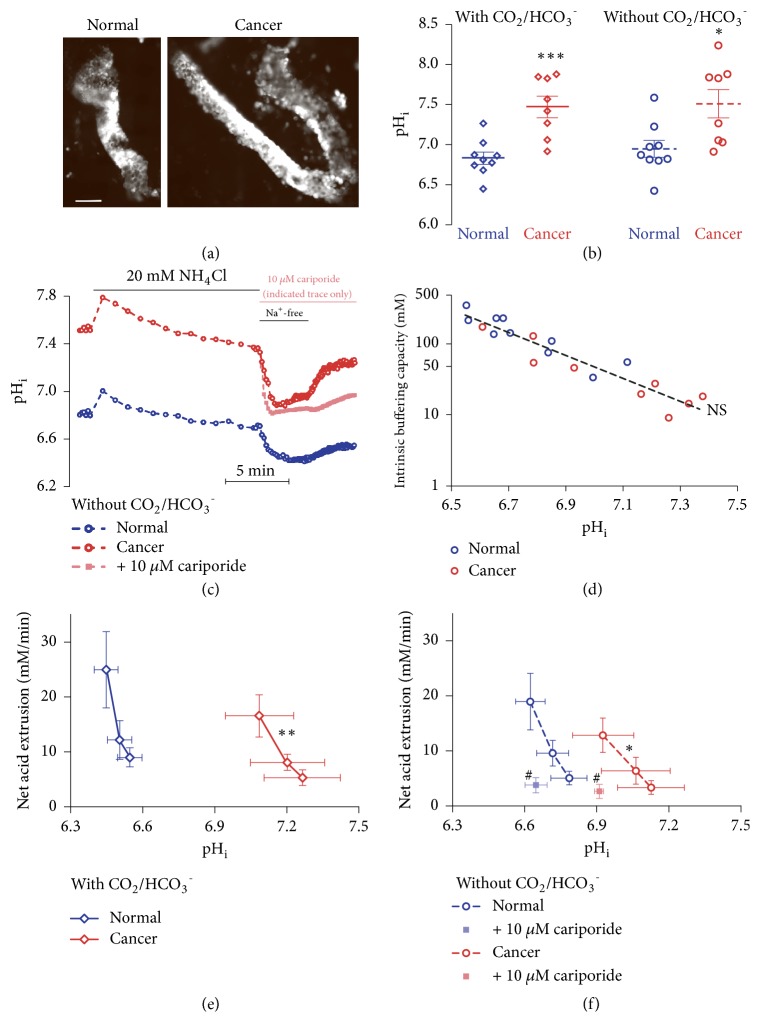
Resting steady-state pH_i_ and the capacity for net acid extrusion are elevated in human colon cancer tissue compared to normal colon tissue due to enhanced Na^+^/H^+^-exchange activity. (a) Images of BCECF-loaded colon crypts (495 nm excitation) isolated from normal colon tissue (left panel) and colon cancer tissue (right panel). The size bar represents 100 *μ*m; both images are shown at the same magnification. (b) Steady-state pH_i_—measured at extracellular pH 7.4—is elevated in colon cancer tissue compared with normal colon tissue (n=8-9) both with and without CO_2_/HCO_3_^–^ present. We compared data by repeated-measures two-way ANOVA followed by Sidak's posttests. (c) Original traces of pH_i_ during NH_4_^+^-prepulse experiments performed in the absence of CO_2_/HCO_3_^–^. In the illustrated experiment performed in presence of 10 *μ*M cariporide, this Na^+^/H^+^-exchange inhibitor was added at the time of NH_4_Cl washout and maintained in the bath solution for the rest of the experiment. (d) Intrinsic buffering capacity is similar in crypts from human colon cancer tissue and normal colon tissue (n=9-11). Based on least-squares linear regression analysis, we compared buffering capacities—plotted on a log-scale—as function of the corresponding pH_i_ values calculated as the mean of the pH_i_ before and after NH_4_Cl washout. Neither slope (*P*=0.80) nor intercept (*P*=0.10) differed significantly between the groups. (e+f) Rates of net acid extrusion plotted as function of pH_i_ in crypts from human colon cancer tissue and normal colon tissue (n=8-11) in the presence (e) and absence (f) of CO_2_/HCO_3_^–^. In the experiments (n=3) performed in presence of 10 *μ*M cariporide, this Na^+^/H^+^-exchange inhibitor was added at the time of NH_4_Cl washout and maintained in the bath solution for the rest of the experiment. We compared lines by least-squares regression analyses and the effect of cariporide in normal and cancer tissue by two-way ANOVA. ^*∗*^*P* < 0.05, ^*∗∗*^*P* < 0.01, and ^*∗∗∗*^*P* < 0.001. NS: not significantly different* vs.* normal tissue under similar conditions. ^#^*P*<0.05* vs.* tissue without cariporide evaluated at similar pH_i_.

In order to circumvent the risk of phenotypical changes occurring during cell culture, we investigated pH_i_ in crypt-like structures (Figure 1(a)) freshly isolated from patients with colon cancer.

### 3.1. Steady-State pH_i_ is Elevated in Colon Cancer Crypts

Steady-state pH_i_ was elevated in crypts from human colon cancer tissue compared with normal colon tissue (Figure 1(b)). The difference in intracellular acidity was evident in the presence as well as in the absence of CO_2_/HCO_3_^–^ (Figure 1(b)). These findings support that enhanced HCO_3_^–^-independent transport processes increase net acid extrusion in the near-neutral pH_i_ range.

### 3.2. Net Acid Extrusion Is Increased in Colon Cancer Crypts

We induced stable intracellular acidification by adding 20 mM extracellular NH_4_Cl and after 15 minutes replacing it by Na^+^-free buffer (Figure 1(c)). From the NH_4_^+^-prepulse-induced intracellular acidification in absence of CO_2_/HCO_3_^–^, we calculated the intrinsic intracellular buffering capacity, which was similar in crypts from human colon cancer and normal colon tissue (Figure 1(d)).

Net acid extrusion was predominantly Na^+^-dependent (Figure 1(c)). In the human colon cancer tissue, 95.5±7.5% and 99.3±6.0% of the overall pH_i_ recovery rate were Na^+^-dependent in the presence and absence of CO_2_/HCO_3_^–^, respectively. Corresponding values for normal colon tissue were 101.1±12.4% and 99.2±7.9%.

The cancer cells were able to eliminate intracellular acid at comparatively higher pH_i_ levels than normal epithelial cells (Figures 1(e) and 1(f)). This finding further emphasizes the upregulated capacity for net acid extrusion in human colon cancer tissue compared to normal colon tissue. Notably, the increased capacity for Na^+^-dependent net acid extrusion both in presence (Figure 1(e)) and in absence (Figure 1(f)) of CO_2_/HCO_3_^–^ supports a key role of Na^+^/H^+^-exchange. This conclusion is further reinforced by the observation that 10 *μ*M of the Na^+^/H^+^-exchange inhibitor cariporide almost entirely blocked net acid extrusion from both human colon cancer and normal colon crypts (Figures 1(c) and 1(f)).

## 4. Discussion

We successfully isolated crypt-like structures from biopsies of human colonic adenocarcinomas and matched normal colon tissue (Figure 1(a)). Based on these freshly isolated tissue preparations, we show that steady-state pH_i_ is dramatically elevated (by ~0.5) in colon cancer tissue compared to normal colon tissue and that this difference in pH_i_ does not depend on CO_2_/HCO_3_^–^ (Figure 1(b)). We also show that Na^+^-dependent net acid extrusion is increased in colon cancer crypts compared with normal colon crypts in the presence as well as in the absence of CO_2_/HCO_3_^–^ (Figures 1(e) and 1(f)). These findings and the efficient inhibition of net acid extrusion achieved upon addition of 10 *μ*M cariporide (Figures 1(c) and 1(f)) support a predominant role of Na^+^/H^+^-exchange for pH_i_ regulation in colon cancer tissue. Working with freshly isolated colonic crypts—rather than cultured cell lines—reduces the risk of changes in cell function and protein expression caused by cell culture and has the advantage of maintained cellular interactions and overall architecture. The polarization of the colonic epithelium is increasingly compromised in dedifferentiated cancers that also gradually loose crypt structure [13]. The relatively well-maintained crypt-like structure of the investigated preparations (Figure 1(a)) supports that upregulation of net acid extrusion is an early carcinogenic event that occurs already in well-differentiated cancer cells.

In congruence with the current study, the capacity for net acid extrusion is much greater in human breast cancer tissue compared to normal breast tissue [14, 15]. However, the molecular mechanism for enhanced acid extrusion in breast carcinomas depends on Na^+^,HCO_3_^–^-cotransport rather than Na^+^/H^+^-exchange [15–17]. Together, these findings highlight that cancer cells rely on enhanced net acid extrusion in order to develop and progress in the acidic tumor microenvironment but the molecular machinery allowing them to do so and hence the most promising targets for anticancer therapy vary between different types of cancers.

It is notable from the current study that the relationship between net acid extrusion and pH_i_ for the normal colon tissue shifts to the left upon addition of CO_2_/HCO_3_^–^ to the bath solution whereas this is not the case for the colon cancer tissue (compare Figures 1(e) and 1(f)). The reason for this phenomenon is not yet clear but it may reflect a much more substantial Cl^–^/HCO_3_^–^-exchange activity in the normal colon tissue compared to the colon cancer tissue. This hypothesis is consistent with earlier reports that the expression of the anion-exchanger SLC26A3 (Down-Regulated in Adenoma, DRA) decreases during colon carcinogenesis [18, 19].

The intrinsic intracellular buffering capacities of human colon cancer tissue (Figure 1(d)) and human breast cancer tissue [15] are similar to those of equivalent normal tissue. Protonatable groups on proteins and peptides are important for intracellular buffering, and the intrinsic buffering capacity will therefore depend on the protein expression patterns of the cells, which may again be determined by the degree of cellular dedifferentiation.

## 5. Conclusions

In conclusion, our experiments—based on freshly isolated crypts from human colon tissue—demonstrate a dramatic increase in Na^+^/H^+^-exchange activity and steady-state pH_i_ in colon cancer tissue compared to normal colon tissue.

## Data Availability

Anonymized data generated and analyzed during this study are available from the corresponding author on reasonable request.

## List of Abbreviations

pH_i_: Intracellular pH.
